# Supplementary material for: Negative reflection of elastic guided waves in chaotic and random scattering media
Source: Sci Rep. 2019 Feb 14;9:2135. doi: 10.1038/s41598-019-38480-3 (PMC6376041; doi:10.1038/s41598-019-38480-3)
Supplement: Supplementary file 1 — Supplementary Material [file 41598_2019_38480_MOESM1_ESM.pdf]

Supplementary Information: Negative reflection of elastic guided waves in chaotic and random scattering media

Benoît Gérardin[1], Jérôme Laurent[1], François Legrand[1], Claire Prada[1], and Alexandre Aubry[1,*]

[1]ESPCI Paris, PSL Research University, CNRS, Univ Paris Diderot, Sorbonne Paris Cité, Institut Langevin, UMR 7587, 1 rue Jussieu, F-75005 Paris, France
[*]alexandre.aubry@espci.fr

ABSTRACT

This document provides further information on the separation of the incident and negatively reflected components of the wave-field recorded in the truncated billiard. It also provides the details of the calculations that lead to its theoretical expression in the monochromatic regime.

S1. Separation of the S_{2b} and S_1 modes in the truncated billiard

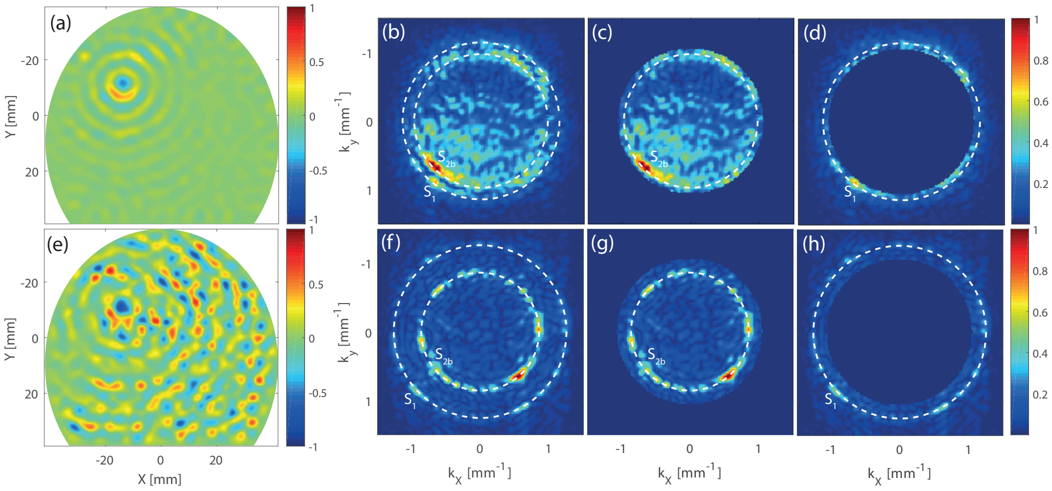

Figure S1. Fourier analysis of the wave-field measured in the truncated billiard at the ZGV frequency $f = 1.91$MHz (a-d) and above ZGV at $f = 1.92$ MHz (e-h). From left to right : the wave fields (a,e), their spatial Fourier transforms (b,f), applied low-pass filter, $|k| < k_{ZGV}$, in order to isolate the S2b contribution (c,g), applied high-pass filter, $|k| > k_{ZGV}$, to isolate the S1 contribution (d,h).

Fig. S1(a) displays the wave-field recorded by the interferometric probe at frequency $f = 1.91$ MHz in the truncated billiard [see Fig. 2(a) of the accompanying paper]. Its spatial Fourier transform is displayed in Fig. S1(b). It exhibits two rings in the vicinity of $k_{ZGV} = 1.06$ mm^{-1}. The inner ring is associated with the S_{2b} mode ($k_{2b} = 0.975$ mm^{-1}) and the outer ring with the S_1 mode ($k_1 = 1.145$ mm^{-1}). To separate both contributions, spatial low and high pass filters are applied with a cut-off at k_{ZGV}. Fig. S1(c) displays the result of the low pass filter in the spatial Fourier domain ($|k| < k_{ZGV}$). An inverse discrete Fourier transform (DFT) then yields the incident S_{2b} wave-field shown in Fig. 3(a) of the accompanying paper. Fig. S1(d) displays the result of the high pass filter in the spatial Fourier domain ($|k| > k_{ZGV}$). An inverse DFT then yields the negatively reflected S_1 wave-field shown in Fig. 3(c) of the accompanying paper. Fig. S1(e) displays the wave-field recorded by the interferometric probe at frequency $f = 1.92$ MHz. Its spatial Fourier transform is displayed in Fig. S1(f). The inner ring is associated with the S_{2b}-mode ($k_{2b} = 0.86$ mm^{-1}) and the outer ring with the S_1-mode ($k_1 = 1.26$ mm^{-1}). To separate both contributions, spatial

low- and high-pass filters are applied with a cut-off at k_{ZGV}. Fig. S1(g) displays the result of the low-pass filter in the spatial Fourier domain ($|k| < k_{ZGV}$). Fig. S1(h) displays the result of the high-pass filter in the spatial Fourier domain ($|k| > k_{ZGV}$). Its inverse DFT then yields the reflected S_1 wave-field shown in Fig. 3(b) of the accompanying paper.

S2. Theoretical expression of the wave-field induced by negative reflection in a cavity

Let us assume a point source at $\mathbf{r_0}$ in a chaotic cavity [see Fig.2(a) of the accompanying paper]. We assume that this source selectively emits a divergent forward wave (*i.e.* of positive phase velocity). This choice is arbitrary and the same final result would be obtained if a divergent backward incident wave was considered. The incident wave-field $\phi_0(\mathbf{r})$ is given by the causal forward Green's function $G_0^+(\mathbf{r}|\mathbf{r_0})$ that would be obtained in free space. A first negative reflection event at the cavity boundary gives rise to a converging backward wave that mathematically corresponds to the anti-causal backward Green's function $G_0^{-*}(\mathbf{r}|\mathbf{r_0})$. As in a time-reversal experiment[1], this wave collapses at the initial source point $\mathbf{r_0}$ and is always followed by a diverging wave. The first order reflected wave-field $\phi_1(\mathbf{r})$ thus shows two wave-fronts, the second one being the conjugate of the first one, but multiplied by -1:

$$\phi_1(\mathbf{r}) = \rho\left[G_0^{-*}(\mathbf{r}|\mathbf{r_0}) - G_0^{-}(\mathbf{r}|\mathbf{r_0})\right] \tag{S1}$$

where the symbol $*$ stands for phase conjugate and ρ is the reflection coefficient. The diverging wave $-\rho G_0^-(\mathbf{r}|\mathbf{r_0})$ in the last equation is one more time negatively reflected. The second order reflected wave-field is then given by:

$$\phi_2(\mathbf{r}) = \rho^2\left[G_0^{+}(\mathbf{r}|\mathbf{r_0}) - G_0^{+*}(\mathbf{r}|\mathbf{r_0})\right] \tag{S2}$$

The negative reflection process can be iterated. The reflected wave-field can be expressed after an even number of reflection events as,

$$\phi_{2n}(\mathbf{r}) = \rho^{2n}\left[G_0^{+}(\mathbf{r}|\mathbf{r_0}) - G_0^{+*}(\mathbf{r}|\mathbf{r_0})\right], \tag{S3}$$

and after an odd number of reflection events as,

$$\phi_{2n+1}(\mathbf{r}) = \rho^{2n+1}\left[G_0^{-*}(\mathbf{r}|\mathbf{r_0}) - G_0^{-}(\mathbf{r}|\mathbf{r_0})\right] \tag{S4}$$

By noting that $G_0^-(\mathbf{r}|\mathbf{r_0}) = G_0^{+*}(\mathbf{r}|\mathbf{r_0})$ for strict negative reflection, the total wave-field $\phi(\mathbf{r})$ in the cavity can be finally expressed as

$$\phi(\mathbf{r}) = \sum_{i=0}^{\infty}\phi_i(\mathbf{r}) = G_0^{+}(\mathbf{r}|\mathbf{r_0}) + \frac{\rho}{1-\rho}\left[G_0^{+}(\mathbf{r}|\mathbf{r_0}) - G_0^{+*}(\mathbf{r}|\mathbf{r_0})\right] \tag{S5}$$

Depending on the sign of ρ, negative reflection can give rise to opposite interference phenomena. A reflection coefficient ρ close to -1 yields the following expression for $\phi(\mathbf{r})$:

$$\lim_{\rho\to-1}\phi(\mathbf{r}) = \mathrm{Re}\left\{G_0(\mathbf{r}|\mathbf{r_0})\right\}. \tag{S6}$$

The destructive interference of the incident and NR waves gives rise to a stationary wave-field that coincides with the real part of the free-space Green's function. In 3D or 2D configurations, this implies a singularity of the wave-field at the source location and a super-focusing ability, since[2]

$$\mathrm{Re}\left\{G_0^{(3D)}(\mathbf{r}|\mathbf{r_0})\right\} = \frac{\cos\left(2\pi\|\mathbf{r}-\mathbf{r_0}\|/\lambda\right)}{4\pi\|\mathbf{r}-\mathbf{r_0}\|}, \tag{S7}$$

and

$$\mathrm{Re}\left\{G_0^{(2D)}(\mathbf{r}|\mathbf{r_0})\right\} = \frac{Y_0(2\pi\|\mathbf{r}-\mathbf{r_0}\|/\lambda)}{4} \underset{\|\mathbf{r}-\mathbf{r_0}\|<<\lambda}{\sim} \frac{1}{2\pi}\left[\ln\left(\frac{\pi\|\mathbf{r}-\mathbf{r_0}\|}{\lambda}\right)+\gamma\right] \tag{S8}$$

with Y_0 the zero-order Bessel function of the second kind and γ the Euler-Mascheroni constant[3].

On the contrary, a reflection coefficient ρ close to 1 yields the following expression for $\phi(\mathbf{r})$ [Eq. (S5)]:

$$\lim_{\rho\to 1}\phi(\mathbf{r}) = \frac{1}{\varepsilon}\mathrm{Im}\left\{G_0(\mathbf{r}|\mathbf{r_0})\right\}, \tag{S9}$$

with $\rho = 1 - \varepsilon$ and $\varepsilon << 1$. The constructive interference of the incident and NR waves gives rise to a resonant amplification of the wave-field at the source location accounted by the factor ε^{-1} in the last equation. As in a time reversal experiment[1], the induced wave-field is given by the imaginary part of the free-space Green's function, which reads, in 2D and 3D, as follows

$$\mathrm{Im}\left\{G_0^{(2D)}(\mathbf{r}|\mathbf{r_0})\right\} = \frac{J_0(2\pi\|\mathbf{r}-\mathbf{r_0}\|/\lambda)}{4} \tag{S10}$$

and

$$\mathrm{Im}\left\{G_0^{(3D)}(\mathbf{r}|\mathbf{r_0})\right\} = \frac{\sin(2\pi\|\mathbf{r}-\mathbf{r_0}\|/\lambda)}{4\pi\|\mathbf{r}-\mathbf{r_0}\|}. \tag{S11}$$

Unlike the real part of $G_0(\mathbf{r}|\mathbf{r_0})$ [Eqs. (S7)-(S9)], its imaginary part does not exhibit any singularity but shows a typical width of the order of $\lambda/2$. For $\rho \sim 1$, negative reflection thus gives rise to a diffraction-limited focal spot at the initial source location.

References

1. de Rosny, J. & Fink, M. Overcoming the diffraction limit in wave physics using a time-reversal mirror and a novel acoustic sink. *Phys. Rev. Lett.* **89**, 124301 (2002).

2. Watanabe, K. *Integral transform techniques for Green's functions. Chapter 2: Green's Functions for Laplace and Wave Equations* (Springer, Cham, Switzerland, 2014).

3. Weisstein, E. W. Bessel function of the second kind. From MathWorld - A Wolfram Web Resource. URL http://mathworld.wolfram.com/BesselFunctionoftheSecondKind.html.
